# Supplementary material for: Sex differences in the applicability of Western cardiovascular disease risk prediction equations in the Asian population
Source: PLoS One. 2024 Jan 31;19(1):e0292067. doi: 10.1371/journal.pone.0292067 (PMC10830057; doi:10.1371/journal.pone.0292067)
Supplement: S1 Table — (DOCX) [file pone.0292067.s001.docx]

**S1 Table. Equation parameters for each model by sex**

|  | **Men** | | | **Women** | | | |
| --- | --- | --- | --- | --- | --- | --- | --- |
|  | **Data-driven (SE)** | **Framingham** | **ASCVD** | | **Data-driven (SE)** | **Framingham** | **ASCVD** |
| Ln age | - | 3.06117 | 12.344 | | - | 2.32888 | -29.799 |
| Ln age square | 0.436 (0.011) | - | - | | 0.463 (0.015) | - | 4.884 |
| Ln total cholesterol | - | 1.1237 | 11.853 | | - | 1.20904 | 13.54 |
| Ln HDL-cholesterol | - | -0.93263 | -7.99 | | -0.186 (0.088) | -0.70833 | -13.578 |
| Ln treated SBP | 2.728 (0.151) | 1.99881 | 1.797 | | 1.757 (0.159) | 2.82263 | 2.019 |
| Ln untreated SBP | 2.513 (0.149) | 1.93303 | 1.764 | | 1.591 (0.157) | 2.76157 | 1.957 |
| Smoking | 0.103 (0.037) | 0.65451 | 7.837 | | - | 0.528873 | 7.574 |
| Diabetes | - | 0.57367 | 0.658 | | - | 0.69154 | 0.661 |
| Ln body mass index | 0.523 (0.158) | - | - | | 0.562 (0.170) | - | - |
| Ln age*Ln total cholesterol | - | - | -2.664 | | - | - | -3.114 |
| Ln age*Ln HDL-cholesterol | - | - | 1.769 | | - | - | 3.149 |
| Ln age* smoking | - | - | -1.795 | | - | - | -1.665 |
| Ln LDL-cholesterol | - | - | - | | -0.325 (0.062) | - | - |
| Baseline survival | 0.961 | 0.88936 | 0.9144 | | 0.954 | 0.95012 | 0.9665 |
| Mean (Coefficient × Value) |  | 23.9802 | 61.18 | | 13.86 | 26.1931 | -29.18 |

SE, standard error; ASCVD, atherosclerotic cardiovascular disease risk equations; HDL, high-density lipoprotein; SBP, systolic blood pressure

“-“ means N/A.
