# Supplementary material for: Sex differences in the applicability of Western cardiovascular disease risk prediction equations in the Asian population
Source: PLoS One. 2024 Jan 31;19(1):e0292067. doi: 10.1371/journal.pone.0292067 (PMC10830057; doi:10.1371/journal.pone.0292067)
Supplement: Attachment — Submitted filename: Response to Reviewers ver.3.docx [file pone.0292067.s002.docx]

**<Response to Reviewers >**

**Thank you very much for your careful review and advice on our research. All the authors highly respect the opinions of the reviewers, and all the authors have discussed deeply and tried to make modifications to reflect all the opinions of the reviewers. This is a study in the process of establishing a new model to confirm the risk of cardiovascular disease using big data of a Korean sample cohort. The revised manuscript was submitted as a track change version and a clean version. Responses are written in blue text color, and actual corrections are written in green text. Please check the revised contents and give a positive review result. Thank you again.**

**General Comment:**

**In your Data Availability statement, you have not specified where the minimal data set underlying the results described in your manuscript can be found. PLOS defines a study's minimal data set as the underlying data used to reach the conclusions drawn in the manuscript and any additional data required to replicate the reported study findings in their entirety. All PLOS journals require that the minimal data set be made fully available. For more information about our data policy, please see** [**http://journals.plos.org/plosone/s/data-availability**](http://journals.plos.org/plosone/s/data-availability)**.**

- **Answer:** Thank you for your comment. We added the ‘Data availability’ section in the manuscript. Public sharing for our raw data is forbidden, and the data access is only allowed to registered researchers.

Therefore, the next contents have been added.

- **Change in the manuscript:**

**Data availability**

Third party data was obtained for this study from National Health Insurance Service-National Sample Cohort (NHIS-NSC), South Korea. Data may be requested from NHIS after creating an account and registering for access. More access information can be found on the NHIS website (https://nhiss.nhis.or.kr). The authors confirm that interested researchers would be able to access these data in the same manner as the authors. The authors also confirm that they had no special access privileges that others would not have.

**Comment (1-2)**

1) Abstract. The introduction is poorly written and needs a very extensive rephrasing in order to provide to the reader a very straightforward message. Actually, there is not a clear rational about the gender-difference that is postulated in the heading and the design of the study and the analysis.
2) Abstract: conclusion section is even more elusive and very poorly clear. At the end the reviewer believes that the reader would not be provided of a clear message from this data. Please, provide a very important revision of the abstract and, consequently, of the rationale in the background/introduction section.

**Answer:** That's an important point. The abstract was completely revised. The results and conclusions are clearly written based on the research contents.

- **Change in the manuscript:**
- Aims: Cardiovascular diseases (CVDs) are the most common cause of death, but they can be effectively managed through appropriate prevention and treatment. An important aspect in preventing CVDs is assessing each individual's comprehensive risk profile, for which various risk engines have been developed. The important keys to CVD risk engines are high reliability and accuracy, which show differences in predictability depending on disease status or race. Framingham risk score (FRS) and the atherosclerotic cardiovascular disease risk equations (ASCVD) were applied to the Korean population to assess their suitability.
- Methods: **A** retrospective cohort study was conducted using National Health Insurance Corporation sample cohort from 2003 to 2015. The enrolled participants over 30 years of age and without CVD followed-up for 10 years. We compared the prediction performance of FRS and ASCVD and calculated the relative importance of each covariate.
- Results: The AUCs of FRS (men: 0.750; women: 0.748) were higher than those of ASCVD (men: 0.718; women: 0.727) for both sexes (Delong test P <0.01). Goodness of fits (GOF) were poor for all models (Chi-square P < 0.001), especially, underestimation of the risk was pronounced in women. When the men’s coefficients were applied to women’s data, AUC (0.748; Delong test P<0.01) and the GOF (chi-square P=0.746) were notably improved in FRS. Hypertension was found to be the most influential variable for CVD, and this is one of the reasons why FRS, having the highest relative weight to blood pressure, showed better performance.
- Conclusion: When applying existing tools to Korean women, there was a noticeable underestimation. To accurately predict the risk of CVD, it was more appropriate to use FRS with men’s coefficient in women. Moreover, hypertension was found to be a main risk factor for CVD.

**Comment (3)**

3) Appendix 1. The descriptive table is not very helpful and actually the reviewer is not convinced about its added value. Vice versa, a table with descriptive statistics of the population strata according to CVD risk would be even more informative. In this way, this could be the first main table starting from the results section.

- **Answer:** Sorry for the uncertainty of the descriptive tables. We agreed that Appendix 2 (descriptive statistics of the population strata according to CVD risk) contains important information. In Appendix 1, We provided additional information by adding P-values generated by the t-test and the chi-square test, indicating the degree of sex difference for each variable. These tables (Appendix 1 and Appendix 2) were moved to the main part (Table 1-2) considering the importance of their contents. We also added the ‘Descript Statistics’ section in the ‘Result’, which provides brief descriptions of these tables.
- **Change in the manuscript:**

**Descriptive Statistics**

Several distinctive features were observed between men and women (Table 1). For example, women (46.3 ± 9.9) were older than men (42.5 ± 10.1), and men had a higher smoking rate (44.8% vs. 1.9%) and higher levels of triglycerides (149.0 ± 97.0 vs. 107.7 ± 64.4). The incidence rates of each subtype of CVD outcomes also varied based on sex (Table 2). Especially, the incidence of I63 (Cerebral infarction), which was the most frequently occurring subtype, was significantly higher in women (327.63 [95% CI, 308.46 to 347.58]) compared to men (240.65 [95% CI, 227.3 to 254.5]).

**Table 1. Baseline characteristics of the study population (Originally: Appendix 1)**

| **Risk factors** | **Men** | **Women** | **P-value** |
| --- | --- | --- | --- |
|  | **(N=50,619)** | **(N=33,468)** |  |
| Age, year (Mean ± SD)  Body Mass Index, kg/m^2^ (Mean ± SD)  Fasting serum glucose, mg/dL (Mean ± SD) | 42.5 ± 10.1  23.7 ± 2.8  92.4 ± 19.4 | 46.3 ± 9.9  22.9 ± 2.9  89.9 ± 17.2 | <0.001  <0.001  <0.001 |
| Total cholesterol, mg/dL (Mean ± SD)  LDL-cholesterol, mg/dL (Mean ± SD) | 193.1 ± 32.3  115.2 ± 35.8 | 191.5 ± 34.0  119.2 ± 34.7 | <0.001  <0.001 |
| HDL-cholesterol, mg/dL (Mean ± SD)  Triglyceride, mg/dL (Mean ± SD) | 51.9 ± 12.3  149.0 ± 97.0 | 58.0 ± 13.0  107.7 ± 64.4 | <0.001  <0.001 |
| Systolic BP, mmHg (Mean ± SD) | 123.4 ± 14.9 | 117.8 ± 16.6 | <0.001 |
| Smoking, *n* (%) | 22659 (44.8%) | 637 (1.9%) | <0.001 |
| Diabetes^a^, *n* (%)  Hypertension^b^, *n* (%) | 1911 (3.8%)  3125 (6.2%) | 1019 (3.0%)  2374 (7.1%) | <0.001  <0.001 |

SD, standard deviation; LDL, low-density lipoprotein; HDL, high-density lipoprotein; BP, blood pressure.

^a^ Diabetes was defined as fasting serum glucose $\geq$126 mg/dL or history of diagnosis (ICD code-E11.9).

^b^ Hypertension was defined as systolic blood pressure of $\geq$140 mmHg or history of diagnosis (ICD code-I10).

**Table 2. Incidence rates of CVD outcomes with person-years and events at 10 years of follow-up in the populations (Originally: Appendix 2)**

| **ICD code** | **Description** | **Men** | |  | **Women** | |
| --- | --- | --- | --- | --- | --- | --- |
|  |  | **Events** | **Incidence rate^a^**  **(95% CI)** |  | **Events** | **Incidence rate^a^**  **(95% CI)** |
| I63 | Cerebral infarction | 1203 | 240.65 (227.3, 254.5) |  | 1078 | 327.63 (308.46, 347.58) |
| I61 | Intracerebral haemorrhage | 125 | 24.73 (20.64, 29.32) |  | 80 | 23.94 (19.07, 29.57) |
| G45 | Transient cerebral ischaemic attacks and related syndromes | 142 | 28.09 (23.72, 32.97) |  | 174 | 52.11 (44.75, 60.24) |
| I50 | Heart failure | 74 | 14.63 (11.54, 18.22) |  | 80 | 23.93 (19.06, 29.56) |
| I48 | Atrial fibrillation and flutter | 541 | 107.44 (98.64, 116.75) |  | 301 | 90.38 (80.55, 100.98) |
| I20 | Angina pectoris | 591 | 117.42 (108.21, 127.14) |  | 395 | 118.73 (107.4, 130.83) |
| I21 | Acute myocardial infarction | 450 | 89.38 (81.38, 97.9) |  | 271 | 81.36 (72.05, 91.43) |
| I60 | Subarachnoid haemorrhage | 62 | 12.26 (9.45, 15.57) |  | 67 | 20.04 (15.61, 25.23) |
| I46 | Cardiac arrest | 56 | 11.07 (8.42, 14.22) |  | 16 | 4.78 (2.8, 7.52) |
| I62 | Other nontraumatic intracranial haemorrhage | 50 | 9.88 (7.39, 12.88) |  | 16 | 4.78 (2.8, 7.52) |

CVD, cardiovascular disease; ICD, International Classification of Diseases; CI, confidence interval

^a^ Incidence rate per 100,000 person-years

**Comment (4)**

4) Table1 and Table 2. The way covariates are presented is not easy-to-get for the readers. Perhaps, some more explanation of how the models are conducted a which is the sense of each factor included in this table would be appreciated.

- **Answer:** Thank you for your comment. We’ve incorporated an explanation of the risk score calculations in the ‘Materials and Methods’ to aid reader comprehension. Table 1, which simply presents the list of parameters used in each model, has been relocated to the Supporting information section (S1 Table).
- Furthermore, we’ve enriched the description of what was previously the Table 2, now Table 4. The model previously known as "KCDRS" has been renamed to "data-driven" to reflect its role more accurately. This ‘data-driven’ model was built to optimally fit our data, and is used as a gold standard to evaluate other models. Both FRS and the data-driven model identified Ln-treated SBP as the most influential variable. Given that the data-driven model highlighted blood pressure as the most critical risk factor in our study population, the model assigning the greatest weight to SBP (FRS) provided the most precise fit to the data.
- **Change in the manuscript:**

**Risk score calculation**

Both the FRS and the ASCVD were developed based on the Cox proportional hazards method, and the features used in them are nearly identical.[6, 13] Equation parameters are listed in S1 Table. For example, CVD risk for men in FRS is calculated as follows:

$L=\beta_{0}\times\ln\left( Age \right)+ \beta_{1}\times\ln\left( Total cholesterol \right)+\beta_{2}\times\ln\left( HDL cholesterol \right)+\beta_{3}\times\ln\left( Systolic blood pressure \right)+\beta_{4}\times Treated for blood pressure+\beta_{5}\times Smoker+ \beta_{6}\times Diabetes-$Mean (*Coefficient × Value*)

$$CVD Risk=1-\left( Baseline survival \right)^{\exp(L)}$$

Values in S1 Table corresponding to each variable should be inserted in each beta.

**Developing the data-driven model**

We developed a data-driven model that exhibited the best performance in our data, with detailed coefficients outlined in S1 Table. The model’s AUC was 0.780 (95% CI, 0.771 to 0.789) for men and 0.776 (95% CI, 0.766 to 0.786) for women. Hosmer-Lemeshow test yielded P-value of 0.003 for men, 0.37 for women, respectively (Fig 4). By developing the data-customized model, we used it as a benchmark for evaluating and comparing pre-existing models. The coefficient that swapped in the FRS coefficients for women – yielding the best prediction among given models – showed performance closest to that of the data-driven model. Although it had a slightly lower AUC, it produced identical results in the GOF test.

**The impact of variables on CVD risk prediction**

To discern which covariate exerted the most significant effect on the outcome, we computed the relative proportion of variances explained by each covariate (Table 4). When employing the FRS, the most critical variable was blood pressure (Ln-treated SBP was 5.785 for men and 15.881 for women); for the ASCVD, the most important variable was smoking for men (14.658) and age for women (21.698). In the data-driven model, treated SBP emerged as the most influential variable for both men (10.891) and women (6.192). The value for ‘Ln-treated SBP’ was the highest in all models except for the ASCVD, indicating that the most influential variable for CVD outcome was consistent in both the FRS and the data-driven models. This alignment substantiates our finding that the FRS demonstrated higher performance and a better fit with our data, which indicates blood pressure was the most significant risk factor in our study population, and the model that attributed the greatest weight to SBP (FRS) best fit our data.

**Table 4. Variance explained by each covariate for Framingham, ASCVD risk score, and the data-driven models (Originally: Table 2)**

|  | **Data-driven model** | | **Framingham** | | | **ASCVD** | | |  |
| --- | --- | --- | --- | --- | --- | --- | --- | --- | --- |
|  | **Men** | **Women** | | **Men** | **Women** | | **Men** | **Women** | |
| Ln age | - | - | | 0.461 | 0.238 | | 3.735 | 21.698 | |
| Ln age square | 0.552 | 0.561 | | - | - | | - | 36.375 | |
| Ln total cholesterol | - | - | | 0.036 | 0.046 | | 3.615 | 5.108 | |
| Ln HDL-cholesterol | - | 0.002 | | 0.048 | 0.026 | | 3.619 | 9.655 | |
| Ln treated SBP | 10.891 | 6.192 | | 5.785 | 15.881 | | 7.219 | 9.94 | |
| Ln untreated SBP | 9.284 | 5.034 | | 5.434 | 15.08 | | 7.003 | 9.309 | |
| Smoking | 0.003 | - | | 0.106 | 0.005 | | 14.658 | 1.107 | |
| Diabetes | - | - | | 0.012 | 0.014 | | 0.022 | 0.015 | |
| Ln body mass index | 0.004 | 0.004 | | - | - | | - | - | |
| Ln age × Ln total cholesterol | - | - | | - | - | | 7.557 | 13.356 | |
| Ln age × Ln HDL-cholesterol | - | - | | - | - | | 3.89 | 10.251 | |
| Ln age × smoking | - | - | | - | - | | 11.564 | 0.823 | |
| Ln LDL-cholesterol | - | 0.009 | | - | - | | - | - | |

ASCVD, atherosclerotic cardiovascular disease risk equations; SBP, systolic blood pressure; HDL, high-density lipoprotein; LDL, low-density lipoprotein.

**^a^** Relative proportion of variances explained by each covariate over the variance of the prediction model.

“-“ means N/A.

**Comment (5)**

5) Figure 2, Figure 3, Table 3. Major point of criticism: actually, the authors are evaluating the areas under the curve of each model and how the performance is, for each single model, but a direct comparison of the models is not provided and, as a result, the authors are not provided about a clear evidence of the effectiveness of one algorithm compared to the other. This point, if is believed to be addressed by authors, should be more significantly emphasized (and better described).

- **Answer:** Thank you for your comment. To more directly illustrate comparisons between each model, we have restructured the ‘Result’ section with a more detailed explanation, and adjusted the tables and figures. In the revised version, both Table 3 and Figure 2 exclusively feature the results derived from the original FRS and ASCVD models. Table 3 displays the AUC values for the two models and the P-values obtained from AUC comparison. Additionally, chi-square values and P-values in Fig 2 come from the goodness of fit test, where a smaller chi-square value and a higher P-value suggest that the model provides a more accurate representation of the observed data. Fig 3 and 4 present consolidated results from both the Delong test and the chi-square test, comparing the original models with the new models. These results facilitate a comprehensive understanding and comparison of which model delivers superior prediction accuracy.
- **Change in the manuscript:**

**Comparison of performance between FRS and ASCVD risk score**

The results of predictability assessment are presented in Table 3. The AUCs of FRS (men: 0.750 [95% CI, 0.741 to 0.760]; women: 0.748 [95% CI, 0.738 to 0.759]) were significantly higher than those of ASCVD (men: 0.718 [95% CI, 0.707 to 0.729]; women: 0.727 [95% CI, 0.715 to 0.738]) for both sexes (P < 0.01).

Fig 2 displays I results of comparing the predicted and actual incidence of CVD using both the FRS and the ASCVD risk score. The overall distributions for both scores were divided into 10 deciles to present the mean predicted score for each interval. Chi-square tests produced P-values less than 0.001, indicating poor GOF for all models. In men, the original FRS model predicted a CVD incidence of 7.93%, while the observed incidence was 6.01%. IThe ASCVD risk score, on the other hand, estimated the CVD incidence at 6.38%, but the observed incidence was 9.47%. In women, the predicted CVD incidence by the original FRS model was 3.90%, whereas the observed incidence was 6.92%. Furthermore, the ASCVD risk score estimated the CVD incidence as 2.23%, while the observed incidence was 8.76%.

These results indicate that the observed incidence of CVD in women was underestimated by both tool when using the original model. Consequently, we decided to apply the men’s coefficients to the women’s data.

**Fig 2.** **Comparison of the calibration by decile between FRS and ASCVD models.** Vertical bars represent observed (black) and predicted (grey) risks.

**Table 3. Comparison of prognostic performance between FRS and ASCVD risk score models**

|  |  | **Framingham** | **ASCVD** |
| --- | --- | --- | --- |
| Men | AUC (95% CI) | 0.750 (0.741-0.760) | 0.718 (0.707-0.729) |
|  | Sensitivity | 63.8 | 61.2 |
|  | Specificity | 75.7 | 71.6 |
|  | P-value | <0.01 ^a^ | |
| Women | AUC (95% CI) | 0.748 (0.738-0.759) | 0.727 (0.715-0.738) |
|  | Sensitivity | 73.0 | 61.7 |
|  | Specificity | 64.6 | 72.8 |
|  | P-value | <0.01 ^b^ | |

FRS, Framingham risk score; ASCVD, atherosclerotic cardiovascular disease risk equations; AUC, area under the curve; CI, confidence interval

^a^ P-values were generated from the Delong test comparing AUCs for Framingham and ASCVD in men

^b^ P-values were generated from the Delong test comparing AUCs for Framingham and ASCVD in women

**Application of men coefficients in women data**

The AUC demonstrated a significant increase from 0.748 (95% CI, 0.738 to 0.759) to 0.755 (95% CI, 0.750 to 0.766) for the FRS (P < 0.01). This improvement was corroborated by a substantial improvement in the GOF test, with the Hosemer-Lemeshow test no longer exhibiting significance (P = 0.746). Conversely, in the case of ASCVD, there was no significant change in the AUC (P = 0.39), and the P-value from the chi-square test was lower than 0.001, indicating persistent underestimation of CVD risk (Fig 3). In summary, the FRS showed a marked improvement in model performance, when compared to the ASCVD.

**Fig 3. Performance of model with men coefficients applied to female data.** Vertical bars represent observed (black) and predicted (grey) risks. P-values from Delong test were used to compare the AUCs generated using the original model coefficients

**Developing the data-driven model**

We developed a data-driven model that exhibited the best performance in our data, with detailed coefficients outlined in S1 Table. The model’s AUC was 0.780 (95% CI, 0.771 to 0.789) for men and 0.776 (95% CI, 0.766 to 0.786) for women. Hosmer-Lemeshow test yielded P-value of 0.003 for men, 0.37 for women, respectively (Fig 4). By developing the data-customized model, we used it as a benchmark for evaluating and comparing pre-existing models. The coefficient that swapped in the FRS coefficients for women – yielding the best prediction among given models – showed performance closest to that of the data-driven model. Although it had a slightly lower AUC, it produced identical results in the GOF test.

**Fig 4. Performance of Data-driven model.** Vertical bars represent observed (black) and predicted (grey) risks. P-values from Delong test were used to compare the AUCs for generated using the original coefficients of the Framingham model.

**Comment (6)**

6) Discussion is too long and there is not a clear presentation and discussion about the limits. Please, revise this aspect.

**Answer:** In the discussion, we identified and described the differences of our tool from existing tools such as FRS and the contents of other studies. In addition, redundant or repetitive background content was resolutely deleted. Some references have been removed. Please review the text. We were impressed by the reviewer's meticulous review. Thank you.
